# Prognostic Value of Iron-Homeostasis Regulating Peptide Hepcidin in Coronary Heart Disease—Evidence from the Large AtheroGene Study

**DOI:** 10.3390/biom8030043

**Published:** 2018-06-28

**Authors:** Tanja Zeller, Alev Altay, Christoph Waldeyer, Sebastian Appelbaum, Francisco Ojeda, Julia Ruhe, Renate B. Schnabel, Karl J. Lackner, Stefan Blankenberg, Mahir Karakas

**Affiliations:** 1 Department of General and Interventional Cardiology, University Heart Center Hamburg, 20246 Hamburg, Germany; t.zeller@uke.de (T.Z.); alevaltay@web.de (A.A.); c.waldeyer@uke.de (C.W.); sebastian.appelbaum@tu-dortmund.de (S.A.); f.ojeda-echevarria@uke.de (F.O.); juleruhe@hotmail.de (J.R.); r.schnabel@uke.de (R.B.S.); s.blankenberg@uke.de (S.B.); 2 German Center for Cardiovascular Research (DZHK), Partner Site Hamburg, Lübeck, Kiel, 20246 Hamburg, Germany; 3 German Center for Cardiovascular Research (DZHK), Partner Site Rhein-Main, 55131 Mainz, Germany; Karl.Lackner@unimedizin-mainz.de; 4 Department of Laboratory Medicine, University Medical Center, Johannes Gutenberg University Mainz, 55131 Mainz, Germany

**Keywords:** hepcidin, iron, coronary heart disease, biomarker, prognosis

## Abstract

Iron is essential in terms of oxygen utilization and mitochondrial function. The liver-derived peptide hepcidin has been recognized as a key regulator of iron homeostasis. Since iron metabolism is crucially linked to cardiovascular health, and low hepcidin was proposed as potential new marker of iron metabolism, we aimed to evaluate the prognostic value of hepcidin in a large cohort of patients with coronary heart disease (CHD). Serum levels of hepcidin were determined at baseline in patients with angiographically documented CHD. The main outcome measure was non-fatal myocardial infarction (MI) or cardiovascular death. During a median follow-up of 4.1 years, 10.3% experienced an endpoint. In Cox regression analyses for hepcidin the hazard ratio for future cardiovascular death or MI was 1.03 (95% confidence interval (CI) 0.91–1.18, *p* = 0.63) after adjustment for sex and age. This association virtually did not change after additional adjustment for body mass index (BMI), smoking status, hypertension, diabetes, dyslipidemia, and surrogates of cardiac function (NT-proBNP), size of myocardial necrosis (troponin I), and anemia (hemoglobin). In this study, by far the largest evaluating the predictive value of hepcidin, hepcidin levels were not associated with future MI or cardiovascular death. This implicates a limited, if any, role for hepcidin in secondary cardiovascular risk prediction.

## 1. Introduction

Iron is a trace element and is essential in terms of oxygen transport, oxygen utilization, and mitochondrial function [1]. Iron metabolism and respective disorders are a major issue in cardiovascular medicine: patients with heart failure (HF) or acute myocardial infarction (MI) and concomitant iron deficiency are at increased risk of future cardiovascular events and see improvement with intravenous iron supplementation, which has emerged as a guideline-endorsed therapy [2,3,4,5].

Hepcidin, which was discovered in the last decade, is a peptide hormone synthesized mainly in the liver [6]. It is now recognized that hepcidin is the key regulator of iron homeostasis in humans, who by nature have no capacity of active iron excretion [6]. The hepcidin production is feedback-regulated by iron levels: in case of iron deficiency or increased iron needs, the resulting suppression of hepcidin levels increases serum iron by elevated iron absorption through enterocytes in the duodenum, and by forced release of iron from macrophages [7].

Since the established factors of iron metabolism—ferritin and transferrin saturation—have already proven predictive value in inflammatory responses, cardiovascular disease, and cardiovascular mortality, the question raised is whether circulating levels of hepcidin might be an even better predictive parameter [2,8,9,10]. Various experimental studies reported strongly raised hepcidin levels during hypoxia, myocarditis, and myocardial ischemia [11,12,13]. Interestingly, while hypoxia appears to induce hepcidin in heart tissue, the opposite is the case in the liver, where hepcidin is suppressed during hypoxia [11]. Likewise, in epidemiological studies, altered hepcidin levels have been linked with metabolic syndrome, arterial hypertension, aortic stiffness, pulmonary arterial hypertension, atherosclerosis, severity of systolic HF, and with poor outcome in patients with stable and acutely decompensated HF [8,14,15,16,17,18,19]. Although results from the aforementioned studies seem to be fairly consistent, discussion on hepcidin and its role in cardiovascular disease remains controversial, since all performed studies had methodological limitations—either due to small sample size, to a cross-sectional design, to testing in primary-prevention settings and interpretation of results in manifest disease settings, or due to missing adjustment for potential confounders.

We sought to elucidate the role of hepcidin in the prediction of cardiovascular death and non-fatal MI, beyond established and emerging cardiovascular risk factors, in a large prospective cohort of patients with coronary heart disease (CHD).

## 2. Materials and Methods

### 2.1. Study Population

A total of 3800 patients, who underwent coronary angiography at the Department of Medicine II of the Johannes Gutenberg University Mainz or the Bundeswehr-Zentralkrankenhaus Koblenz, were recruited in the AtheroGene Study between June 1999 and March 2004 [20]. The exclusion criteria were evidence of haemodynamically significant valvular heart disease, surgery or trauma within the previous month, known cardiomyopathy, known cancer, febrile conditions, or use of oral anticoagulant therapy within the previous 4 weeks. Subjects with missing information on the clinical presentation, or missing information on the cause of death were additionally ruled out, resulting in 3423 patients with CHD. After inobservance of subjects with missing samples or low sample volume, measurement of hepcidin was performed in 2198 patients. There were no relevant differences in baseline characteristics between the subcohort and the overall CHD cohort (data not shown).

All subjects gave written informed consent. The study was performed in accordance with the Declaration of Helsinki and approved by the Ethics Board of the Johannes Gutenberg University Mainz and of the Physicians’ chamber of the State Rhineland-Palatinate (Mainz, Germany) under the ethical number 837.057.99.

### 2.2. Data Collection

At baseline, all participants were subjected to a standardized questionnaire containing socio-demographic information and medical history. In addition, information was taken from the patients’ hospital charts. Coronary artery disease (CAD) was diagnosed if the coronary angiogram showed at least one stenosis >30% in a major coronary artery. Acute coronary syndrome (ACS) comprised unstable angina and acute myocardial infarction (AMI). Unstable angina was diagnosed according to Braunwald criteria [21]. AMI was either ST-segment elevation with significant elevation (STEMI) in at least two contiguous leads or non-ST-elevation myocardial infarction (NSTEMI) based on clinic and positive in-house troponin concentrations. Status of haemochromatosis was not assessed.

In all patients, active follow-up was performed until a median of 4.1 years after discharge. Information was obtained from the patients using a mailed standardized questionnaire. Information regarding adverse cardiovascular disease (CVD) events and treatment since discharge from the in-hospital rehabilitation clinic was obtained from the primary care physicians also by means of a standardized questionnaire. If a subject had died during follow-up, the death certificate was obtained from the local Public Health Department and the main cause of death was coded according to the International Classification of Diseases (ICD-9 pos. 390–459: ICD-10 pos. I0-I99 and R57.0). Adverse CVD events were defined as CVD as the main cause of death (as stated in the death certificate).

### 2.3. Laboratory Methods

At baseline, blood was drawn before angiography in a fasting state under standardized conditions and stored at −80 °C until analysis. Serum hepcidin was measured using the Hepcidin 25 (bioactive) HS ELISA from DRG [13]. The inter- and intra-assay coefficient of variation was 9.7% and 8.7%, respectively. These measurements were performed from prior unthawed aliquots. C-reactive protein (CRP), N-terminal pro brain natriuretic peptide (NT-proBNP), troponin I, total cholesterol, high-density lipoprotein-cholesterol (HDL), and low-density lipoprotein-cholesterol (LDL) cholesterol measurements were done by routine methods in the participating hospitals. All biomarkers were measured in a blinded fashion.

### 2.4. Statistical Methods

The study population was described with respect to various sociodemographic and medical characteristics.

Cardiovascular death and non-fatal MI were defined as the outcome measures. The relation of hepcidin with (i) cardiovascular mortality and/or non-fatal MI, and (ii) cardiovascular morality only, during follow-up was assessed by Cox regression analyses adjusted for age, sex in model 1, and additionally for hypertension, smoking status, diabetes, hyperlipidemia, body mass index (BMI), hemoglobin, log (NT-proBNP), and log (troponin I) in model 2.

All statistical procedures were carried out using R 3.2.4 (http://www.r-project.org/). A *p*-value < 0.05 was considered as statistically significant.

## 3. Results

**Figure 1:**
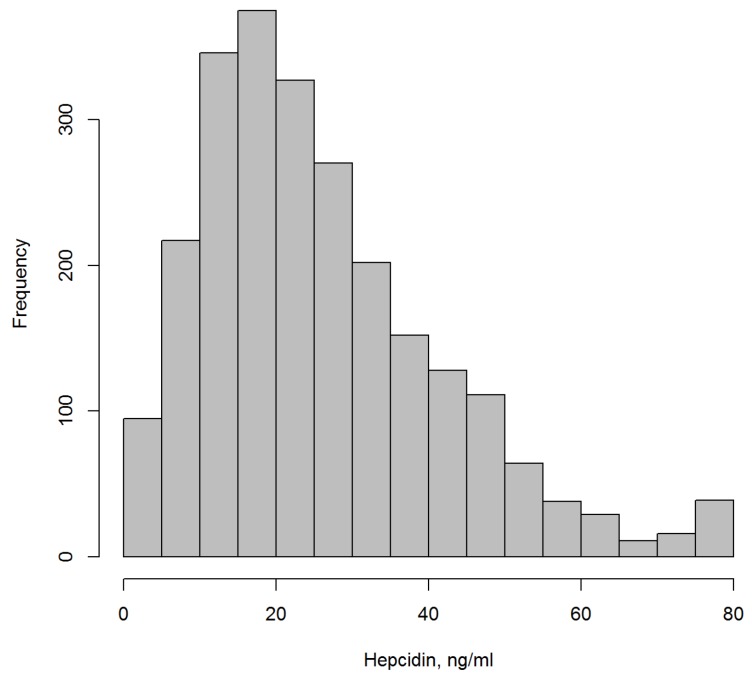
Distribution of hepcidin levels in the AtheroGene Study (*n* = 2198).

Table 1 shows the main sociodemographic and laboratory characteristics at baseline. Mean age was 63.0 years, and participants were predominantly men (75.2%). Median hepcidin was 22.8 ng/mL (25/75 percentiles: 14.2; 34.5). Figure 1 shows the distribution of hepcidin levels.

During a median follow-up of 4.1 years, 10.3% of patients experienced cardiovascular death and/or non-fatal MI. In Cox regression analyses the hazard ratio (HR) for future cardiovascular death or MI per one SD increase of hepcidin was 1.03 after adjustment for sex and age ((95% CI 0.91–1.18), *p* = 0.63) (Table 2). This association virtually did not change after additional adjustment for BMI, smoking status, hypertension, diabetes, dyslipidemia, and surrogates of cardiac function (N-terminal pro B-type natriuretic peptide), size of myocardial necrosis (troponin I), and anemia (hemoglobin) (HR 0.95 (95% CI 0.79–1.14), *p* = 0.57).

Cox regression analyses regarding cardiovascular mortality only (without non-fatal MI) during 4.1-years follow-up also did not yield significant association (Table 3): the HR for future cardiovascular death per one SD increase of hepcidin was 1.04 after adjustment for sex and age ((95% CI 0.87–1.25), *p* = 0.65), and 0.88 ((95% CI 0.69–1.12), *p* = 0.29) in a fully adjusted model.

## 4. Discussion

Iron metabolism and respective disorders are of utmost importance in cardiovascular medicine. It is now recognized that the liver-derived peptide hepcidin is the key regulator of iron homeostasis in humans and mammals, and recent studies, mostly performed in primary-prevention settings, have highlighted hepcidin as most auspicious new marker of iron metabolism and its dysfunction in cardiovascular disease.

We sought to elucidate the role of hepcidin in the prediction of cardiovascular death and non-fatal MI, beyond established and emerging cardiovascular risk factors, in a large prospective cohort of patients with CHD. Of note, the diagnosis of CHD was exclusively based on coronary angiography.

We could not find any independent association between hepcidin levels and future risk of MI or cardiovascular death—neither in a basic model adjusted for age and sex, nor in a more comprehensively adjusted model. This is in contrast to previous, much smaller reports, and implicates a limited, if any, role for hepcidin in secondary cardiovascular risk prediction. Despite pathophysiological implications, we could not demonstrate any additional value for hepcidin in individuals with manifest CHD.

Experimental studies have suggested that hepcidin might mirror hypoxia and ischemia: in rat heart, hypoxia results in a strong upregulation of hepcidin expression on mRNA and protein level, accompanied by an increased immunoreactivity of hepcidin pronounced at the myocardial intercalated disc area [11]. These findings were confirmed in a rat model of acute MI and extended to patients with acute myocarditis: hepcidin showed an abrupt increase of up to 100 times in human cardiomyocytes within one day after myocardial infarction and acute myocarditis [12,13]. Moreover, the receptor for hepcidin, ferroportin, is not just highly expressed in duodenal enterocytes, but also in reticuloendothelial macrophages, which themselves are involved in all stages of atherogenesis [7,22].

The discrepancy between our results and previous reports might arise from the fact that we studied the prognostic value of hepcidin in a cohort with existing cardiovascular disease, while experimental evidence for hepcidin mirroring hypoxia and ischemia comes from primary disease settings. This is in line with epidemiological studies, which were mostly performed in primary- prevention or in community-based settings, and which reported hepcidin to be associated with predictors of cardiovascular disease and very early stage of cardiovascular disease, like arterial hypertension, metabolic syndrome, aortic stiffness, or other measures of subclinical atherosclerosis [17,18,19]. Additionally, it might be that circulating hepcidin levels measured in the present study do not mirror changes in cardiac hepcidin levels associated with CHD. As hepcidin is primarily expressed in the liver most of the circulating hepcidin in blood is derived from the liver and not the heart.

## 5. Conclusions

In this study, by far the largest evaluating the predictive value of hepcidin in patients with manifest CHD, levels of hepcidin were not associated with future MI or cardiovascular death. This implicates a limited, if any, role for hepcidin in secondary cardiovascular risk prediction. Based on findings from other epidemiological studies, we strongly emphasize evaluation of hepcidin as marker of incident CHD in primary-prevention settings.

## Figures and Tables

**Table 1:** Baseline characteristics of the study patients.

*n*	3423
Age (years) *	63.0 (55.0, 69.8)
Male sex (%)	75.2
BMI (kg/m^2^) *	27.0 (24.9, 29.7)
Current smoker (%)	27.8
Diabetes (%)	21.6
Hypertension (%)	74.4
Hyperlipidemia (%)	67.7
History of MI (%)	40.9
Total Cholesterol (mg/dL) *	205.0 (175.7, 235.0)
HDL-C (mg/dL) *	47.0 (39.0, 57.0)
LDL-C (mg/dL) *	127.0 (102.0, 155.0)
NT-proBNP (pg/mL) *	226.2 (96.0, 659.1)
CRP (mg/dL) *	3.6 (1.6, 9.9)
Hemoglobin (g/dL)*	14.4 (13.4, 15.2)
Hepcidin (ng/mL) *	23.2 (15.4, 34.7)

BMI = body mass index, HDL-C = high-density lipoprotein-cholesterol, LDL-C = low-density lipoprotein-cholesterol, CRP = C-reactive protein, MI = myocardial infarction, NT-proBNP = N-terminal pro brain natriuretic peptide, sTfR = soluble transferrin receptor. * Median 25th and 75th quartile cut-point.

**Table 2:** Association of hepcidin per one SD increase with CV death and nonfatal MI during 4 years of follow-up.

	HR	95% CI	*p*-Value
Model 1	1.03	0.91–1.18	0.63
Model 2	0.95	0.79–1.14	0.57

SD = standard deviation, CV = cardiovascular, HR = hazard ratio, CI = confidence interval. Model 1: adjusted for age and sex. Model 2: Model 1 additionally adjusted for hypertension, smoking status, diabetes, hyperlipidemia, BMI, hemoglobin, log (NT-proBNP), log (troponin I).

**Table 3:** Association of hepcidin per one SD increase with CV death during 4 years of follow-up.

	HR	95% CI	*p*-Value
Model 1	1.04	0.87–1.25	0.65
Model 2	0.88	0.69–1.12	0.29

Model 1: adjusted for age and sex. Model 2: Model 1 additionally adjusted for hypertension, smoking status, diabetes, hyperlipidemia, BMI, hemoglobin, log (NT-proBNP), log (troponin I).
